# Unveiling the Public Economic Burden of Migraine in Argentina

**DOI:** 10.36469/001c.133639

**Published:** 2025-04-09

**Authors:** Zrinka Orlović, Lucila Rey-Ares, María Florencia Viozzi, Rui Martins, Juliana Villarreal Ramírez, Santiago Veiga, Mark P. Connolly

**Affiliations:** 1 Global Market Access Solutions SARL, Chardonne, Switzerland; 2 Faculty of Economics and Business, Department of Managerial Economics, University of Zagreb, Zagreb, Croatia; 3 Pfizer SRL, Villa Adelina, Argentina; 4 University Medical Center Groningen, Department of Health Sciences, Unit of Global Health University of Groningen, Groningen, Netherlands; 5 Pfizer SAS, Bogotá, Colombia; 6 Global Market Access Solutions SARL, Chardonne, Switzerland

**Keywords:** economic burden, migraine, Argentina, work productivity, absenteeism, informal employment, healthcare costs

## Abstract

**Background:** Migraine is a prevalent, underdiagnosed, highly debilitating neurological condition that affects individuals’ quality of life and often negatively influences normal daily activities. **Objectives:** The study objective is to estimate the economic burden of migraine to the Argentine government by assessing the impact of the disease on tax revenue, absenteeism, and social support transfers. **Methods:** The analysis combines a cross-sectional model utilizing national demographic data and published migraine prevalence rates to estimate the annual burden for the entire migraine-affected cohort, and a longitudinal model assessing the average burden per individual from the age of 40, over a 20-year horizon. A fiscal framework based on generational accounting evaluated the impact of migraine on government finances. Sources of revenue such as direct and indirect taxes were weighted against elements of public expenditure (public sector absenteeism, healthcare expenses, and financial support) and compared with the general population. The effect of migraine on occupational outcomes was sourced from peer-reviewed publications, and costs were sourced from national databases. Results were reported as incremental fiscal consequences (2023 US dollars) and were discounted at 3% annually. **Results:** The fiscal burden of migraine in Argentina was estimated to be 6505perindividualand1237 million across the entire migraine population. Annually, 29% of government costs were due to public sector absenteeism, 39% related to healthcare costs, 19% to foregone direct and indirect tax revenue, and 12% to foregone corporation taxes. Additional government transfers represented a minor contribution to the overall fiscal impact of migraine in Argentina. **Discussion:** The high rate of informal employment is likely to undermine disease burden estimates. Gender disparities were notable, with women bearing 76% of the burden, highlighting the need for gender-specific interventions. **Conclusions:** This study reveals a significant economic burden of migraine to the Argentinian government, primarily driven by absenteeism, healthcare costs, and foregone tax contributions. Targeted, gender-responsive healthcare and labor policies, especially for sectors with high informal employment, could help reduce these fiscal impacts.

## BACKGROUND

Migraine is a neurological condition that is often underdiagnosed despite deeply affecting individuals’ quality of life.^1^ Migraine is classified into episodic migraine (EM) and chronic (CM) migraine: EM is defined as having up to 14 headache days per month, while CM is diagnosed in individuals experiencing more than 14 headache days per month.^2^ Migraine prevalence varies across countries, with a global prevalence estimated at approximately 15%.^3^ Of relevance to this study, in Argentina, recent publications indicate that overall migraine prevalence is 9.5%, ranging from 6.3% to 12% across different regions.^4^

Pain caused by migraine can vary from moderate to severe and is often accompanied by symptoms such as nausea, vomiting, photophobia, and phonophobia. Episodes can last hours to several days (4-72 hours), worsening with movement and physical activity, consequently impairing daily functioning and quality of life.^5–8^ Migraine, particularly CM, often occurs simultaneously with other conditions. There is a strong association between migraine and depression/anxiety, with depression increasing the risk of migraine to become chronic. Additionally, migraine is associated with other chronic pain issues, like neck and low back pain. Cardiovascular risks, such as ischemic heart disease and stroke, are more significant in patients with migraine with aura. Other related comorbidities include epilepsy and obesity in CM patients. Generally, comorbidities are more common in CM than EM and can contribute to its chronic progression.^9–13^

The burden of migraine falls disproportionately on women, who represent over 75% of all cases. The disease is most frequently developed around the age of 20 years and peaks at 39 years old, which exacerbates the disease’s impact on women during their most active and productive years.^4,14,15^ In a recent large-scale international survey, Argentinian migraine patients reported that migraine disrupted their daily activities in 97% of cases and negatively affected their social life and activities (81%). Notably, nearly 80% of respondents acknowledged a detrimental impact on their professional life, with 56% missing at least 1 workday in the past month due to migraine.^16^ Obtaining a formal diagnosis and access to the appropriate treatment still represent significant challenges for these patients, despite migraine motivating frequent medical consultations and visits to the emergency room every year.^16,17^

Despite the high prevalence of this condition and the considerable public health burden it represents, migraine remains underfunded, underrecognized, and stigmatized.^18^ Acute health events such as migraine shape the choices people make throughout their lifetime, impacting their decisions and translating to quantifiable economic losses.^19^ Migraine-related disability and impact increase with increased headache day frequency among those with EM, with the greatest burden on average reported by individuals with CM.^20^ Migraine is associated with significantly higher absenteeism and productivity lost,^20^ as well as disability, employment, and early retirement.^19,21^

Argentina’s healthcare system comprises 3 subsectors: public universal healthcare, Social Security (union-run health insurance institutions and the National Institute of Social Services for Retirees and Pensioners [PAMI]), and private medical insurance. According to the last census, 17.8 million people (39%) rely exclusively on public health care, especially infants and young adults.^22,23^

In the United States, migraine has been linked to a substantial economic burden, including direct medical and nonmedical costs, as well as indirect costs. People living with migraine have been estimated to incur $6575 more in direct healthcare expenses than individuals unaffected by the condition and to experience more frequent and prolonged work loss, resulting in significantly higher indirect costs.^24,25^ This burden is especially pronounced in individuals with CM, who are more severely affected than those with EM.^26^

When these characteristics are taken into account, migraine would have a substantial burden on society, not only via increased direct costs, but mainly due to indirect costs such as reduced labor participation, absenteeism, and productivity losses. To facilitate standard high-quality care and promote access to migraine-related health and treatment services, evidence-based decision-making is needed for priority setting, and health programs need to be planned based on the local burden of migraine data. The goal of this study is to utilize existing evidence of the effect of migraine on labor participation and an establish fiscal framework to estimate the economic consequences of migraine for the Argentinian government (ie, fiscal burden).^27^

## METHODS

The objective of this study was to combine cross-sectorial data sources to estimate the economic burden of migraine falling on the Argentine government. The analysis adapts a published study conducted from the UK public sector perspective capturing individual governmental transactions based on disease burden applied within an age-specific framework.^28^ All calculations were implemented in Microsoft Excel. Costs were adjusted to reflect 2023 values. An exchange rate of 1 US dollar to 376 Argentine pesos was applied.

### Fiscal Framework

The fiscal framework for this study is grounded in generational accounting, a method widely used by governments to model the economic impact of diseases.^29^ This approach assesses how migraine affects government finances through its influence on employment and productivity. Specifically, it examines whether the disease leads to temporary work absences or, in severe cases, forces individuals to leave the labor market entirely due to disability. Reduced productivity in both the private and public sectors directly impacts tax revenue, diminishing government income. For individuals who experience disability due to migraine, government spending increases due to social transfer payments, such as unemployment or disability benefits. Additionally, the model considers public health and social care expenses associated with migraine. Despite the model’s focus on the public economic burden of migraine, private healthcare and productivity costs estimated in intermediate calculations were also reported. These would be relevant under a societal perspective of costs and are illustrative of the overall burden of migraine on society.

### Modeling Approach

The analysis utilized two modeling approaches (longitudinal and cross-sectional) to estimate the economic burden of migraine. The longitudinal model follows individuals from age 40^30^ over a 20-year work-life expectancy estimating the average cost per person with prevalent migraine. This approach reveals the economic impact on an individual, capturing changes over time and providing insight into the progression of costs related to absenteeism, taxation loss, and public healthcare expenses.

The cross-sectional model estimates the economic burden of the entire population affected by migraine in a single year (2023), estimating the burden across all ages. This model uses demographic data and migraine prevalence rates by age, enabling a comparison between a cohort affected by migraine and a cohort without migraine (general population). Results from the fiscal perspective include public sector absenteeism, impact on taxation, public healthcare costs, and transfers such as unemployment benefits, disability pensions, and old-age pensions. Estimates of gross income losses for private and public sector employees, along with a breakdown of healthcare costs by payer type (societal costs), were reported for completeness.

Unless stated otherwise, subsequent sections describe the base case model parameterization and the implementation of modeling assumption. Additional details on inputs and sources can be found in the **Supplementary Material**.

### Migraine Prevalence and Disease Severity

Age-specific data on migraine prevalence were unavailable in the literature. Consequently, values of 5% for males and 14% for females^4^ were applied in the calculations.

Migraine severity is commonly defined using 2 categories: episodic and chronic. In the literature, EM is present in individuals having up to 14 headache days per month, while CM is diagnosed in people suffering more than 14 headache days per month.^2,31^ In the analysis, 94.6% of individuals were modeled as having EM and 5.4% as having CM.^30^ The average monthly headache days (MHDs) used were 8.2 for EM and 20.2 for CM.^32^

### Absenteeism

Absenteeism data were used to estimate the impact of migraine on work productivity across 3 main types of employment: self-employed, private sector, and public sector. For self-employed individuals, a day lost to migraine translated directly into a reduction in earnings, due to inability to work. For private sector employees, absenteeism due to migraine lead to reduced productivity and potential profit losses for employers, translating into reduced corporation tax. In the public sector, absent employees still receive their salary, albeit the work goes unaccomplished, reflecting a direct loss to the government. Absenteeism was implemented as a percentage of workdays missed from full- or part-time equivalents in the general population (4.2%),^33^ and in the population affected by EM (8.8%) and CM (23.4%).^20^

### Healthcare Costs

Mean annual healthcare costs associated with migraine vary depending on migraine severity and incurred MHDs. For individuals with EM, mean annual healthcare costs were estimated to be $921, while for those with CM costs were estimated to be $2041. These values were calculated as the weighted average of annual resources used and their respective unit costs. It was assumed that only 50% of the migraine population sought medical care,^34^ impacting the overall healthcare cost estimate. Since this model reflects public expenditures, a government healthcare financing rate of 29% was applied.^35^

### Socioeconomic and Demographic Data

Age-and sex-specific population data were available by age, spanning from 0 to 100 years, and sex.^36^ The total population was categorized as economically active or inactive, with data collected in 5-year age groups by sex. The economically active population consisted of individuals who were employed or unemployed.^37^ Employed individuals were classified as employees or self-employed being full-time or part-time workers.^38^ Employees worked in either the public or private sectors.^39^ The economically inactive population considered in this analysis included individuals who were disabled,^40^ early retirees, and regular retirees.^41^

### Impact of Migraine on Labor Outcomes

A literature review was conducted using Embase and PubMed to identify studies reporting relative measures of migraine’s impact on labor participation. Google Scholar was also searched for additional relevant studies. It was aimed to compare individuals with migraine with both the general population and those with less severe migraine; therefore, individuals with EM were assumed to resemble the general population, with a focus on capturing the specific impact of CM. Due to a lack of relative risk data specific to Argentina, data most closely aligned with Argentina’s context were selected. Consequently, the effects of migraine on employment, disability, and retirement were drawn from studies conducted in several countries. All inputs extracted and used in the model are provided in **Table 1,** and additional details regarding the targeted literature search are available in the **Supplementary Material**.

**Table 1.** Impact of Migraine Severity on Labor Outcomes

**Outcome**	**Migraine Severity**	**RR**	**SE**	**Source**
Current employment (full- or part-time)^a^	EM (≤14 MHDs) [reference]	1		Oliveira et al (2023),^42^ Brazil
	CM (>14 MHDs) [RR]	0.984	0.013	Oliveira et al (2023),^42^ Brazil
Full-time/part-time ratio^b^	CM vs EM	0.963	0.040	IBMS Study, multicountry (Blumenfeld et al [2011]^43^ and Payne et al [2011])^44^
Disability^c^	CM vs EM	1.802	0.084	Oliveira et al (2023),^42^ Brazil
Early retirement^d^	CM vs EM	1.611		IBMS Study, multicountry (Blumenfeld et al [2011]^43^ and Payne et al [2011])^44^

Abbreviations: CM, chronic migraine; EM, episodic migraine; IBMS, International Burden of Migraine study; MHDs, monthly headache days; RR, relative risk; SE, standard error.

^a^It was assumed that individuals with EM would behave like the general population. Relative risk calculated as the ratio of CM-employed individuals to active people vs EM-employed individuals to active people: (242/314)/(5772/7207).

^b^Relative risk calculated as the ratio of people with CM in full-time employment to people with EM in full-time employment: (174/237)/(4033/5291).

^c^Calculated as the ratio of chronic to episodic proportions of people with disability: 20.0%/11.1%.

^d^Calculated as the ratio of proportions of early retirement, CM to EM: 33.5%/20.8%.

### Earnings and Social Benefits

Earnings were applied to individuals employed in either the private or public sector, with the assumption that employees and self-employed individuals receive similar wages. In the private sector, part-time employees are assumed to earn 50% of full-time wages, with data segmented by age groups and sex across life stages, from 18 to 65 years and older. Public sector earnings followed a similar structure, assuming the same relationship between part-time and full-time earnings as in the private sector. Data informing unemployment benefits were not age- or sex-specific. In Argentina, only 2.57%^40^ of the unemployed population receives these benefits. The annual value of unemployment benefits applied in the model is $4979.^40^ Disability benefits, similarly, were not age- or sex-specific and were applied to all eligible individuals regardless of demographic factors. The annual value of disability benefits used in the model is $2362.^45^ Retired individuals, whether early or regular retirees, were entitled to $5592 annually.^45^

### Taxes

The model incorporated various tax rates, including the corporate tax rate, value-added tax, and a tax wedge representing personal income tax. A conservative approach was taken by assuming the lowest corporate tax rate of 25% to account for corporate earnings.^46^ The value-added tax was set at 21%,^46^ reflecting the standard Argentine rate on goods and services. A tax wedge of 33.7%^47^ was applied to represent the effective income tax burden on individuals. Notably, pensions in Argentina are not subject to taxation.^48^

### Inflation and Discounting

The Argentine economy has been subject to high inflation rates. Accordingly, costs and wages were adjusted to 2023 values using the recent consumer price index and wage index. In the longitudinal model, costs were discounted at 3% annually.^49^

All model inputs described above inform the base case. Detailed information on these inputs, specifically migraine prevalence and severity, absenteeism, healthcare costs, socioeconomic and demographic data, labor impact, earnings and social benefits, tax rates, and inflation and discount rates, are detailed in the **Supplementary Material**.

### Model Results

Results were reported as incremental fiscal consequences (IFC), calculated as the difference in net present values between the cohort of individuals affected by migraine and the general population without migraine. The net present values for each cohort were computed as the sum of discounted taxes minus transfers over time. Taxes included direct taxes on employment earnings, indirect taxes, and social security contributions, while transfers encompassed financial support and public healthcare costs.

One-way sensitivity analyses were conducted by varying in turn each mean base case input according to its 95% confidence interval. The results were ranked from most to least influential and summarized in tornado diagrams. The base case reports results for cohorts with EM and CM distributions mimicking the general Argentinian population. In addition, 2 scenario analyses were conducted to estimate the average burden associated with hypothetical cohorts composed exclusively by individuals with EM or CM so that the burden associated to disease severity was disaggregated.

A 2-way sensitivity analysis was also conducted, varying the age at model start (longitudinal model) over the model’s time horizon. The results were compiled into a heat map, illustrating the average economic burden of migraine for different ages of onset across an individual’s working life expectancy. Please refer to additional results in the **Supplementary Material**.

## RESULTS

### Longitudinal Results

**Table 2** provides an overview of the IFC and life-years associated with migraine, estimated using a longitudinal model beginning at age 40 and spanning a 20-year time horizon. This model presents per capita costs, discounted at an annual rate of 3.0%. Results reveal that migraine imposes a significant economic burden on Argentina, primarily through lost productivity, reduced tax revenues, and increased public healthcare costs. On average, individuals with migraine were estimated to earn approximately $2753 less in gross income than those in the general population, leading to a decline in both direct and indirect tax contributions. Specifically, 36.93% of this fiscal impact stems from lost public sector productivity, 31.72% from reduced tax revenues, 30.68% from added healthcare costs, and 1.15% from increased government transfers. Overall, the cumulative fiscal burden to the Argentine government due to migraine is estimated at $6505 per person, or $476 per life-year, compared with those without migraine. For individuals with CM, this cost rises to $21 670 per person over the 20-year time horizon.

**Table 2.** Base Case Results of the Longitudinal Model Comparing the Migraine Population and the General Population in Argentina^a^

	**Migraine Population**	**General Population**	**Incremental**	**Fiscal Impact**
Gross income from any employment^b^	$138 648	$141 401	-$2753	
Fiscal consequence				
Public sector absenteeism	-$4275	-$1873	-$2402	36.93% (loss)
Direct taxes from employment	$46 724	$47 652	-$928	14.26% (loss)
Indirect taxes from employment	$19 304	$19 687	-$383	5.89% (loss)
Foregone corporation taxes	-$1339	-$587	-$753	11.57% (loss)
Unemployment allowances	-$84	-$83	-$1	0.02% (loss)
Early retirement pension	-$562	-$543	-$19	0.29% (loss)
Disability pension	-$1277	-$1222	-$55	0.84% (loss)
Taxes from transfers	$807	$776	$31	0.48% (gain)
Healthcare costs	-$1996	$0	-$1996	30.68% (loss)
Total	$57 303	$63 808	-$6505	
Life-years	13.666	13.666	0.000	
Incremental costs per life-year lived with migraine	-$476			

^a^All government costs and tax revenue are discounted at a rate of 3.0%. Negative values indicate financial losses, while positive values reflect revenue sources for the Argentine government.

^b^Foregone earnings from employment constitute losses to public and private sectors and are reported for completeness. These are intermediate values used to calculate direct and indirect taxes paid to the government.

### Cross-Sectional Results

**Table 3** compares the fiscal impact of migraine between the migraine population and the general population in Argentina, as estimated by the cross-sectional model. The results indicate that migraine significantly impacts the Argentine economy, primarily through reduced gross income, decreased tax revenue, increased healthcare costs, and public sector absenteeism. Annual gross earnings from employment in the entire population affected by migraine were estimated to be $24 459 million, $504 million lower than the general population. This income disparity contributes to an overall decline in direct taxes ($170 million) and indirect taxes ($70 million), representing 13.74% and 5.68% of total fiscal losses, respectively. Additional fiscal consequences include increased public sector absenteeism costs adding to $648 million in the migraine population, $364 million more than the general population, and accounting for 29.41% of fiscal losses. Migraine was also associated with a $144 million reduction in corporation taxes due to foregone private sector productivity. Furthermore, migraine-related healthcare costs were estimated to be $478 million, contributing 38.66% to the fiscal burden. Our cross-sectional model suggests that the incremental public cost per life-year affected by migraine in 2023 is $378 and $1237 million across the nationwide migraine population.

**Table 3.** Base Case Results of the Cross-Sectional Model Comparing the Migraine Population and the General Population in Argentina^a^

	**Migraine Population**	**General Population**	**Incremental**	**Fiscal Impact**
Gross income from any employment^b^	$24 459 M	$24 963 M	-$504 M	
Fiscal consequence				
Public sector absenteeism	-$648 M	-$284 M	-$364 M	29.41% (loss)
Direct taxes from employment	$8243 M	$8413 M	-$170 M	13.74% (loss)
Indirect taxes from employment	$3405 M	$3476 M	-$70 M	5.68% (loss)
Foregone corporation taxes	-$256 M	-$112 M	-$144 M	11.64% (loss)
Unemployment allowances	-$24 M	-$23 M	$0 M	0.02% (loss)
Early retirement pension	-$255 M	-$246 M	-$8 M	0.68% (loss)
Disability pension	-$168 M	-$161 M	-$7 M	0.58% (loss)
Taxes from transfers	$1462 M	$1457 M	$5 M	0.41% (gain)
Healthcare costs	-$478 M	$0	-$478 M	38.66% (loss)
Total	$11 282 M	$12 519 M	-$237 M	
Life-years	3 274 692	3 274 692	0.000	
Incremental costs per life-year lived with migraine	-$378			

Abbreviation: M, million.

^a^All government costs and tax revenue are discounted at a rate of 3.0%. Negative values indicate financial losses, while positive values reflect revenue sources for the Argentine government.

^b^Foregone earnings from employment constitute losses to public and private sectors and are reported for completeness. These are intermediate values used to calculate direct and indirect taxes paid to the government.

### The Informal Sector

Informal workers, typically outside the formal tax system, are likely to also be affected by migraine, creating unrecorded fiscal gaps. Breaking down income loss by employment type (see **Supplementary Material**), self-employed individuals, who are often informal workers,^50^ may experience a 93.6% income loss per capita compared with the population unaffected by migraine, in contrast to private and public employees, who face losses of 3.4% and 3.0%, respectively. The predicted overall income loss for the migraine population across all employment types is 29.1%. Healthcare costs further illustrate the burden on informal workers, who, with limited access to employer-sponsored healthcare, rely on out-of-pocket spending which was estimated to account for 37.7% of the societal healthcare losses due to migraine.

### One-Way Sensitivity Analysis

**Figure 1.**
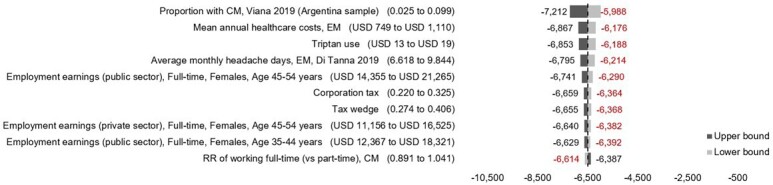
One-Way Sensitivity Analysis of Key Drivers of the Fiscal Impact of Migraine in Argentina (Longitudinal Model) Abbreviations: CM, chronic migraine; EM, episodic migraine; RR, relative risk; USD, US dollars.

A 1-way sensitivity analysis for longitudinal model (**Figure 1**) highlights the primary drivers affecting base case fiscal results per individual. The proportion of individuals with CM influenced results the most, with a potential fiscal range from -$7212 to -$5988. Other influential parameters include mean annual healthcare costs associated with EM, triptan medication use, and the number of MHDs, each of which affect the average economic burden of migraine per person in Argentina. The 1-way analysis suggests these are likely to vary base case estimates by a maximum of ±6%.

### Scenario Analysis

The scenario analysis in **Table 4** examines the impact of migraine severity on base case estimates. As anticipated, modeling exclusively individuals with CM leads to a 91.68% and 81.49% increase in fiscal consequences in the longitudinal and cross-sectional models, respectively. This increase was projected to equate to a cost of $5964 per individual over a 20-year period or $1008 million for the entire population aged 18 to 65. Conversely, shifting the model to assume all individuals are affected by EM yields a 14.2% decrease in the fiscal burden of disease in the longitudinal model and 67.83% in the population model. This reduction represents savings of $924 per individual and a total of $839 million for the entire population. These findings highlight the significant economic implications of migraine severity on fiscal outcomes, with CM posing a much higher cost burden than EM.

**Table 4.** Scenario-Based Fiscal Outcomes: Comparison With Base Case^a^

**Scenario**	**Base Case**		**No CM (100% EM)**		**No EM (100% CM)**	
**Model**	**Longitudinal**	**Population**	**Longitudinal**	**Population**	**Longitudinal**	**Population**
Gross income from any employment^b^	-$2753	-$504 M	-$2157	-$398 M	-$12 469	-$2245 M
Fiscal consequence	-$2402	-$364 M	-$2045	-$310 M	-$8236	-$1248 M
Public sector absenteeism	-$928	-$170 M	-$727	-$134 M	-$4202	-$756 M
Direct taxes from employment	-$383	-$70 M	-$300	-$55 M	-$1736	-$313 M
Indirect taxes from employment	-$753	-$144 M	-$641	-$123 M	-$2580	-$494 M
Foregone corporation taxes	-$1	$0 M	$0	$0 M	-$20	-$4 M
Unemployment allowances	-$19	-$8 M	$0	$0 M	-$332	-$150 M
Early retirement pension	-$55	-$7 M	$0	$0 M	-$980	-$129 M
Disability pension	$31	$5 M	$0	$0 M	$559	$91 M
Taxes from transfers	-$1996	-$478 M	-$1869	-$448 M	-$4143	-$993 M
Healthcare costs	-$2753	-$504 M	-$2157	-$1069 M	-$21 670	-$3995 M
Total	-$6505	-$1237 M	-$5581	-$398 M	-$12 469	-$2245 M
Change from base case			-14.2%	-67.83%	91.68%	81.49%

Abbreviations: CM, chronic migraine; EM, episodic migraine.

^a^Negative monetary values represent financial losses, while positive values indicate revenue sources for the Argentine government. A negative percentage signifies a reduction compared to the base case, while a positive percentage reflects an increase.

^b^Foregone earnings from employment constitute losses to public and private sectors and are reported for completeness. These are intermediate values used to calculate direct and indirect taxes paid to the government.

**Figure 2.**
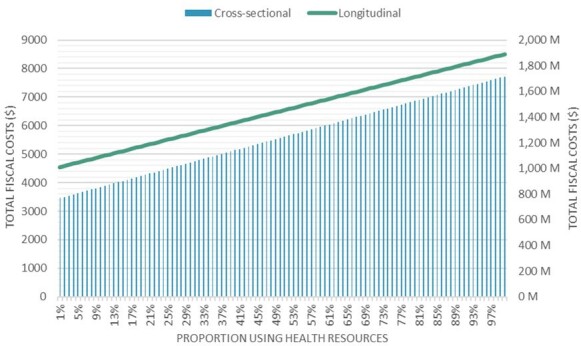
Total Fiscal Costs of Cross-Sectional and Longitudinal Models Based on Proportion of Health Resource Use Abbreviation: M, million.

We have accessed uncertainty around the proportion of individuals accessing healthcare to manage their migraine. Projections for the full range of possible values are shown in **Figure 2**. If 25% of individuals accessed formal healthcare, the total IFC would be $998 million for the entire population, or $5507 per individual. In this scenario, healthcare costs would account for 23.96% of the total burden at the population level and 18.12% of the average individual’s total fiscal burden. If 75% of individuals utilized healthcare resources, the total IFC would rise to $1476 million, or $7503 per individual. Healthcare costs in this case would represent 48.60% of the total burden for the entire population and 39.90% of the average individual’s total fiscal burden.

## DISCUSSION

The model reveals that the estimated total economic burden of migraine in Argentina for 2023 amounts to $6505 per person, or $476 per life-year. Across the entire migraine-affected population, this translates to $1237 million or $378 per life-year lived with the condition. In the longitudinal model, 30.68% of the total burden is assigned to healthcare costs, while the remaining 69.32% is due to productivity loss and foregone tax revenues. The cross-sectional model shows similar results where healthcare costs represent 31.06% of the burden, with the remainder attributed to productivity loss and reduced tax revenues. The similarity between the cross-sectional and longitudinal models reinforces the view that migraine imposes significant economic challenges on both an individual and national level. This notable impact is primarily driven by lost productivity in the public sector, inferred from frequent migraine-related absences. Reduced tax revenues from individual and corporate taxes lead to additional pressure on government budgets, competing with other streams of public spending and services. Additionally, increased government spending on transfers and pensions contributes to the fiscal impact, providing financial support for those with severe migraines who may struggle to maintain full employment. Although smaller contributions come from unemployment allowances, early retirement pensions, disability pensions, and taxes from transfers, the primary costs of migraine fall on productivity loss, reduced tax revenues, and healthcare expenses. Similar results can be found worldwide, where migraine imposes a significant economic burden.^51–56^

An important finding of this analysis is the potential hidden impact of migraine on informal employees. Informal employment refers to jobs that are not registered within official government systems, often meaning that workers are deprived of social security, healthcare benefits, and other formal protection mechanisms,^52^ which may exacerbate the burden of migraine. In Argentina, approximately 50% of workers are thought to be employed informally, and therefore earnings are not subject to tax at source tax deductions.^53^ Heavy losses among self-employed informal workers, as shown in the analysis, underscore the challenge of unrecorded tax contributions and hidden productivity losses due to untracked absenteeism in informal sectors. Additionally, many informal workers, lacking healthcare coverage, rely on the public healthcare system, further amplifying the economic impact. This creates a dual burden—both a loss of tax contributions and increased public healthcare expenditures—intensifying the overall cost of migraine on the economy.

Reliance on national employment statistics will likely undermine productivity losses and informal employment may contribute to lower medical coverage and increased out-of-pocket healthcare expenses, which are not fully captured by this analysis. Ultimately, the above complicates the government’s efforts to maintain sustainable social welfare systems. With a large portion of the workforce unregistered, the fiscal burden is likely not accurately reflected in this study, as formal sector contributions alone must shoulder the economic impact while the informal sector remains largely unaccounted for in tax revenue losses. Additionally, informal workers with migraine may have to bear the full cost of treatment out-of-pocket, potentially leading to lower treatment adherence and increased absenteeism, further compounding productivity losses. The results indicate a significant sex disparity in the fiscal burden of migraine, with women bearing 75.6% of the total impact. This finding reflects the higher prevalence of migraine among women and its potential to further precipitate sex-related inequalities.^54^

These results highlight the need for targeted interventions to reduce absenteeism, improve access to effective treatments, and support the migraine-affected population in the workforce. For instance, a migraine education and management program has been shown to reduce absences by 53% and improve productivity on days when employees experience migraine attacks.^55^ Research also indicates that after a correct diagnosis, migraine-specific therapies significantly improve worker productivity, with most studies finding a statistically significant reduction in productivity loss compared with control groups.^56^ Previous studies have shown that these actions reduce the frequency and severity of migraines, leading to a lower healthcare burden.^57^ Recognition of migraine as a severe condition is crucial to addressing the consequences experienced by those affected. Migraine is a common and disabling neurological disorder that impacts many aspects of daily life. Despite its high prevalence and burden, it remains underdiagnosed, partly due to the stigma surrounding the disease.^58^

Given Argentina’s high rate of informal employment, such interventions must also consider the unique challenges faced by informal workers, potentially extending healthcare access and support programs to those outside the formal workforce. Considering the disproportionate burden on women, it is essential for policy measures to include gender-specific strategies. Such efforts could help reduce the financial burden on public resources, enhance quality of life, and further reduce productivity losses, ultimately improving outcomes for individuals affected by migraine.

The limitations of this analysis can be described in a few key segments. Migraine prevalence, being one of the most important inputs, is essential for accurately estimating the fiscal impact. In our analysis, we used migraine prevalence data sourced from the Argentinian literature, applying it as a single rate for each gender across all ages. The use of a single, sex-specific prevalence rate, without age differentiation, may not fully capture the actual distribution of migraine across different age groups. While this approach reflects the best available data, it would be more accurate to have age-specific prevalence rates for both males and females. Since we found no relative risk data specific to Argentina describing the impact of migraine on labor outcomes, we used data from studies conducted in other countries. In doing so, we assumed that the effect of migraine on fiscal consequences would be similar across countries. Argentina has a large proportion of informal employment, meaning many workers do not have formal contracts and do not contribute to taxes. In the context of this analysis, absenteeism in the public sector for these workers is typically not recorded, leading to gaps in capturing productivity losses. Additionally, informal workers often lack healthcare insurance, increasing their out-of-pocket healthcare costs.

All the mentioned limitations affect the estimations, introducing potential variability in the outcomes. As a result, the estimated fiscal burden may be either underestimated or overestimated, depending on the extent to which these limitations influence the analysis. However, considering all factors included in the model, the results obtained provide a comprehensive reflection of the migraine burden from the perspective of the Argentinian government.

## CONCLUSION

This study illustrates the substantial economic burden of migraine on the Argentinian government, with significant fiscal implications stemming from public sector absenteeism, healthcare expenses, and reduced tax revenues. Absenteeism, which significantly affects productivity, accounted for the largest portion of the total burden, ranging from 30% to 35%, with foregone tax revenue accounting for approximately 31%. Despite being the focus of most economic evaluations, healthcare costs were estimated to represent the remaining 30% to 40% of the losses associated with migraine.

Informal employment is likely to bias estimates of the total burden of migraine, as it limits the visibility of taxable income and productivity losses. Informal workers, which represent a significant portion of the labor force, are likely to be more exposed to out-of-pocket healthcare expenditures and may benefit substantially from targeted policy interventions.

This analysis indicates that the burden of migraine is particularly burdensome in females and people affected by CM, which reinforces the need for targeted healthcare strategies to mitigate these effects. Improved access to effective treatments and comprehensive occupational/health policies could potentially alleviate some of the important economic burden demonstrated in this analysis.

## Supplementary Material

Online Supplementary Material
