# Supplementary material for: Unveiling the Public Economic Burden of Migraine in Argentina
Source: J Health Econ Outcomes Res. 2025 Apr 9;12(1):129–37. doi: 10.36469/001c.133639 (PMC11991664; doi:10.36469/001c.133639)
Supplement: Online Supplementary Material [file jheor_2025_12_1_133639_275872.pdf]

Journal of Health Economics and Outcomes Research

Online Supplementary Material

Unveiling the Public Economic Burden of Migraine in Argentina. *JHEOR*. 2025;12(1):129-137.
doi:10.36469/jheor.2025.133639

Table S1: Percentage Distribution of Economic Activity Status Among Across Age Groups for Males and Females 3

Table S2: Percentage Distribution of Employment Type (Employees vs Self-employed) Across Age Groups for Males and Females 3

Table S3: Percentage of Full-time vs Part-time Employment Across Age Groups for Males and Females 4

Table S4: Distribution of Public Sector Employment and Public Health and Social Care Employment Across Age Groups for Males and Females 4

Table S5: Population Distribution and Prevalence of Retirement or Pension Receipt Across Age Groups for Males and Females 4

Table S6: Age Distribution and Prevalence of Disabled Receiving Disability Benefits Across Age Groups for Males and Females 5

Table S7: PubMed Literature Search Strategy (English)Publications in Spanish and Portuguese 7

Table S8: PubMed Literature Search Strategy Filtering for Publications in Spanish and Portuguese 7

Table S9: Embase Literature Search Strategy (English) 7

Table S10: Embase Literature Search Strategy Filtering for Publications in Spanish and Portuguese 8

Figure S1: Literature Search Flow Diagram 8

Table S11: Average Annual Earnings by Age, Employment Type, Sector, and Sex 10

Table S12: Annual Absenteeism Rates for the General and Migraine Populations 10

Table S13: Mean Annual Healthcare Resource Utilization and Local Unit Costs for Migraine 11

This is an open-access article distributed under the terms of the Creative Commons Attribution 4.0 International License (CCBY-4.0). View this license's legal deed at http://creativecommons.org/licenses/by/4.0 and legal code at http://creativecommons.org/licenses/by/4.0/legalcode for more information.

Table S14: Healthcare Financing by Source Type 11

Table S15: Annual Consumer Price Index (CPI) and Wage Index 11

Table S16: Societal Impact of Migraine on Employment Income and Healthcare Costs for an Average Individual (Longitudinal Model) 12

Table S17: Societal Impact of Migraine on Employment Income and Healthcare Costs for the Overall Population (Cross-Sectional Model) 12

Figure S2: Two-Way Sensitivity Analysis of the Average Economic Burden of Migraine by Age of Onset and Time Horizon (Longitudinal Model, in Thousand US Dollars) 13

References 14

This supplementary material has been provided by the authors to give readers additional information about their work.

1. Argentina Labor Market Participation

1.1. Argentina Active Population

Table S1 – Percentage Distribution of Economic Activity Status Among Across Age Groups for Males and Females

	Age Categories	All[a]		Economically Active	
		Economically Active	Economically Inactive	Employed	Unemployed
Males	15 to 19	6.95%	93.05%	84.07%	15.93%
	20 to 24	29.68%	70.32%	78.91%	21.09%
	25 to 29	75.87%	24.13%	85.81%	14.19%
	30 to 34	87.75%	12.25%	92.62%	7.38%
	35 to 39	90.93%	9.07%	95.24%	4.76%
	40 to 44	91.86%	8.14%	96.23%	3.77%
	45 to 49	91.67%	8.33%	96.41%	3.59%
	50 to 54	90.96%	9.04%	96.42%	3.58%
	55 to 59	88.36%	11.64%	96.15%	3.85%
	60 to 64	82.80%	17.20%	95.81%	4.19%
	65 to 69	72.16%	27.84%	95.58%	4.42%
	70 to 74	47.08%	52.92%	95.64%	4.36%
	75 to 79	30.13%	69.87%	95.85%	4.15%
	80 and over	21.51%	78.49%	95.89%	4.11%
Females	16 to 19	5.49%	94.51%	79.55%	20.45%
	20 to 24	22.86%	77.14%	67.79%	32.21%
	25 to 29	62.18%	37.82%	76.86%	23.14%
	30 to 34	73.32%	26.68%	85.98%	14.02%
	35 to 39	76.51%	23.49%	89.67%	10.33%
	40 to 44	77.67%	22.33%	91.26%	8.74%
	45 to 49	77.64%	22.36%	92.16%	7.84%
	50 to 54	76.48%	23.52%	93.08%	6.92%
	55 to 59	72.07%	27.93%	93.67%	6.33%
	60 to 64	62.47%	37.53%	94.04%	5.96%
	65 to 69	40.25%	59.75%	94.30%	5.70%
	70 to 74	24.10%	75.90%	94.19%	5.81%
	75 to 79	14.56%	85.44%	93.88%	6.12%
	80 and over	9.64%	90.36%	93.05%	6.95%

Source: INDEC. Population aged 14 years and over in private households, by economic activity status, according to sex registered at birth and five-year age groups (2022).[1]

[a] Individuals over the age of 70 are assumed to be economically inactive.

Table S2 presents data on employees and self-employed individuals, categorized by age and sex. The 'employees' category includes those in domestic service and employee or worker, while 'self-employed' encompasses all self-employed individuals and employers.

Table S2 - Percentage Distribution of Employment Type (Employees vs Self-employed) Across Age Groups for Males and Females

Age Categories	Employees	Self-employed	Employees	Self-employed
	Males		Females	
15 to 24	67.4%	32.6%	69.5%	30.5%
25 to 54	63.5%	36.5%	69.6%	30.4%
55 to 74	43.4%	56.6%	58.8%	41.2%

Source: INDEC. Employed population aged 14 years and over in private households, by occupational category, according to sex registered at birth and five-year age groups (2022) [2]

Table S3 was calculated based on the reported number of individuals by the number of hours worked per week. It was assumed that all individuals working fewer than 35 hours per week were part-time employed, while those working more than 35 hours were considered full-time.

Table S3 - Percentage of Full-time vs Part-time Employment Across Age Groups for Males and Females

Age Categories	Full-Time[b]	Part-Time	Full-Time	Part-Time
	Males		Females	
15 to 24	66.9%	33.1%	37.8%	62.2%
25 to 34	80.8%	19.2%	47.7%	52.3%
35 to 44	84.1%	15.9%	47.4%	52.6%
45 to 54	81.1%	18.9%	47.1%	52.9%
55 to 64	73.0%	27.0%	46.6%	53.4%
65 and over	59.5%	40.5%	39.5%	60.5%

Source: INDEC. Employed Population - Number of hours worked in the week in the main occupation by age and gender.[3]

[b] Full-time vs part-time was assumed to be the same for employees and self-employed.

Table S4 – Distribution of Public Sector Employment and Public Health and Social Care Employment Across Age Groups for Males and Females

Age Categories	Public Sector Employees [a]	Public Health and Social Care [b]	Public Sector Employees	Public Health and Social Care
	Males		Females	
15 to 24	15.3%	10.0%	28.2%	15.0%
25 to 34	21.5%	12.6%	42.1%	14.8%
35 to 44	24.4%	13.3%	46.7%	14.8%
45 to 54	26.7%	13.2%	48.2%	14.1%
55 to 64	32.3%	13.0%	51.8%	12.6%
65 and over	32.3%	13.0%	86.4%	7.6%

Source: INDEC. Remuneration of salaried work, mixed income and labour input, by sex and age groups (2016-2022).[4]

[a] Calculated as percentage of all employees.

[b] Calculated as percentage of all public employees.

1.2. Argentina Inactive Population

The inactive population consists of individuals who are not employed and are not actively seeking employment. In this model, the inactive population includes those who retire early, those who retire upon reaching retirement age, and those who are unable to work due to disabilities.

Early and regular retirement

Table S5 – Population Distribution and Prevalence of Retirement or Pension Receipt Across Age Groups for Males and Females

Age Categories	Population by Receipt of Retirement or Pension[5]		Argentina Population[6]		Prevalence of Individuals Who Receive Retirement or Pension[a]	
	Males	Females	Males	Females	Males	Females
15 to 19	2,900	2,818	1,800,681	1,768,387	0.2%	0.2%
20 to 24	8,918	9,569	1,757,472	1,779,791	0.5%	0.5%
25 to 29	11,925	12,744	1,755,496	1,824,075	0.7%	0.7%
30 to 34	10,857	10,510	1,706,782	1,787,492	0.6%	0.6%
35 to 39	10,664	9,669	1,616,211	1,692,147	0.7%	0.6%
40 to 44	13,820	12,142	1,617,796	1,713,874	0.9%	0.7%
45 to 49	20,119	15,850	1,386,629	1,488,369	1.5%	1.1%
50 to 54	40,921	39,129	1,177,301	1,281,024	3.5%	3.1%
55 to 59	87,300	97,038	1,044,857	1,158,048	8.4%	8.4%
60 to 64	175,405	526,681	929,041	1,057,693	18.9%	49.8%
65 to 69	493,701	691,395	796,143	946,014	62.0%	73.1%
70 to 74	540,145	557,661	627,993	799,212	86.0%	69.8%
75 to 79	365,199	368,675	424,945	611,035	85.9%	60.3%
80 and over	325,029	344,135	427,838	830,877	76.0%	41.4%

Source: INDEC. Total population in private households. Population receiving retirement or pension according to sex registered at birth and five-year age groups (2022)[5]; INDEC. Total population, by sex registered at birth and femininity index, according to age (2022).[6]

[c] Calculated by dividing number of individuals receiving retirement or pension and total Argentina population by each age category.

Disability

Table S6 – Age Distribution and Prevalence of Disabled Receiving Disability Benefits Across Age Groups for Males and Females

<table>
<tr><th rowspan="2">Age Categories</th><th colspan="2">Argentina Population[6]</th><th colspan="2">Age Distribution Until Retirement Age[a]</th><th colspan="2">Total Disabled Receiving Disability Benefits[b]</th><th colspan="2">Prevalence Of Disabled Receiving Disability Benefits[c]</th></tr>
<tr><th>Males</th><th>Females</th><th>Males</th><th>Females</th><th>Males</th><th>Females</th><th>Males</th><th>Females</th></tr>
<tr><td>15 to 19</td><td>1,800,681</td><td>1,768,387</td><td>6.1%</td><td>6.0%</td><td>69978</td><td>68723</td><td rowspan="3">3.9%</td><td rowspan="3">3.9%</td></tr>
<tr><td>20 to 24</td><td>1,757,472</td><td>1,779,791</td><td>6.0%</td><td>6.1%</td><td>68299</td><td>69166</td></tr>
<tr><td>25 to 29</td><td>1,755,496</td><td>1,824,075</td><td>6.0%</td><td>6.2%</td><td>68222</td><td>70887</td></tr>
<tr><td>30 to 34</td><td>1,706,782</td><td>1,787,492</td><td>5.8%</td><td>6.1%</td><td>66329</td><td>69465</td><td rowspan="2">3.9%</td><td rowspan="2">3.9%</td></tr>
<tr><td>35 to 39</td><td>1,616,211</td><td>1,692,147</td><td>5.5%</td><td>5.8%</td><td>62809</td><td>65760</td></tr>
<tr><td>40 to 44</td><td>1,617,796</td><td>1,713,874</td><td>5.5%</td><td>5.9%</td><td>62871</td><td>66605</td><td rowspan="2">3.9%</td><td rowspan="2">3.9%</td></tr>
<tr><td>45 to 49</td><td>1,386,629</td><td>1,488,369</td><td>4.7%</td><td>5.1%</td><td>53887</td><td>57841</td></tr>
<tr><td>50 to 54</td><td>1,177,301</td><td>1,281,024</td><td>4.0%</td><td>4.4%</td><td>45752</td><td>49783</td><td rowspan="2">3.9%</td><td rowspan="2">3.9%</td></tr>
<tr><td>55 to 59</td><td>1,044,857</td><td>1,158,048</td><td>3.6%</td><td>4.0%</td><td>40605</td><td>45004</td></tr>
<tr><td>60 to 64</td><td>929,041</td><td>1,057,693</td><td>3.2%</td><td>-</td><td>36104</td><td>-</td><td rowspan="2">2.1%</td><td rowspan="2">-</td></tr>
<tr><td>65 to 69</td><td>796,143</td><td>946,014</td><td>-</td><td>-</td><td>-</td><td>-</td></tr>
<tr><td>70 to 74</td><td>627,993</td><td>799,212</td></tr>
<tr><td>75 to 79</td><td>424,945</td><td>611,035</td></tr>
<tr><td>80 and over</td><td>427,838</td><td>830,877</td></tr>
</table>

Source: INDEC. Total population, by sex registered at birth and femininity index, according to age (2022).[6]

[a] The calculation was based on the total population up to the retirement age (65 for males and 60 for females, Source: [7]). After reaching retirement age, individuals begin receiving old-age pensions and stop receiving disability benefits.

[b] Using age distribution data and the total number of disability benefit recipients (1,138,091, Source: The National Social Security Administration [8]), the number of people receiving disability benefits by age and gender was estimated.

[c] Calculated by dividing number of individuals receiving disability benefits and total Argentina population by each age category.

2. Targeted Literature Search

The targeted literature search was conducted on both PubMed and Embase in August 2023 using the migraine mesh heading and terms specific to labor market participation. The search was restricted to English-language articles published within the last 12 years, and European publications were excluded. Searches were also conducted to include relevant publications in Spanish and Portuguese. Resulting titles and abstracts were screened by a single analyst to ensure relevance, with full texts inspected and manually checked for additional references. Studies reporting on relative measures of the impact of migraine on labor participation, compared to the general population or individuals with less severe migraine, were extracted and incorporated into the model.

Table S7 - PubMed Literature Search Strategy (English)

No.	Search Terms	Hits
**1**	(((((("career mobility"[MeSH Terms] OR "caregiver burden"[MeSH Terms] OR "cost of illness"[MeSH Terms] OR "family leave"[MeSH Terms] OR "financial stress"[MeSH Terms] OR "retirement"[MeSH Terms] OR "return to work"[MeSH Terms] OR "social security"[MeSH Terms] OR "social welfare"[MeSH Terms] OR “unemployment”[MeSH Terms]) OR ("Activity impairment"[Title/Abstract] OR "Benefit payment"[Title/Abstract] OR “Compensation”[Title/Abstract] OR "Cost of illness"[Title/Abstract] OR "Disability allowance*"[Title/Abstract] OR Earning*[Title/Abstract] OR “Fiscal”[Title/Abstract] OR "Government transfer"[Title/Abstract] OR "Hidden cost*"[Title/Abstract] OR Income[Title/Abstract] OR "Indirect cost*"[Title/Abstract] OR "Indirect healthcare cost*"[Title/Abstract] OR "Living allowance*"[Title/Abstract] OR "Long-term disability"[Title/Abstract] OR "Lost time"[Title/Abstract] OR "Multifactor productivity"[Title/Abstract] OR Pension*[Title/Abstract] OR "Productive efficiency"[Title/Abstract] OR Retire[Title/Abstract] OR Retirement[Title/Abstract] OR Salaries[Title/Abstract] OR Salary[Title/Abstract] OR "Social benefit*"[Title/Abstract] OR "social insurance benefit"[Title/Abstract] OR "Social security"[Title/Abstract] OR "Tax credit"[Title/Abstract] OR "Total factor productivity"[Title/Abstract] OR "Transfer payment"[Title/Abstract] OR Transportation[Title/Abstract] OR Underemployment[Title/Abstract] OR "Under-employment"[Title/Abstract] OR Unemployment[Title/Abstract] OR "Un-employment"[Title/Abstract] OR Wage[Title/Abstract] OR Wages[Title/Abstract] OR Welfare[Title/Abstract] OR WPAI[Title/Abstract] OR "work life"[Title/Abstract] OR "workers compensation"[Title/Abstract] OR "workers comp"[Title/Abstract])) OR (((work*[Title/Abstract] OR job[Title/Abstract] OR occupation*[Title/Abstract] OR vocation*[Title/Abstract] OR employ*[Title/Abstract] OR workforce[Title/Abstract] OR "work-force"[Title/Abstract] OR "labor force"[Title/Abstract] OR "labour force"[Title/Abstract])) AND (Loss[Title/Abstract] OR productivity[Title/Abstract] OR disability[Title/Abstract] OR participation[Title/Abstract] OR cessation[Title/Abstract] OR status[Title/Abstract] OR outcome[Title/Abstract] OR impair*[Title/Abstract] OR disrupt*[Title/Abstract] OR incapacity[Title/Abstract] OR incapability[Title/Abstract] OR activity[Title/Abstract] OR transportation[Title/Abstract] OR leaving[Title/Abstract]))) OR ((caregiver) AND (work*[Title/Abstract] OR job[Title/Abstract] OR occupation*[Title/Abstract] OR vocation*[Title/Abstract] OR employ*[Title/Abstract] OR workforce[Title/Abstract] OR "work-force"[Title/Abstract] OR "labor force"[Title/Abstract] OR "labour force"[Title/Abstract]))) OR ((fewer[Title/Abstract] OR loss[Title/Abstract] OR lost[Title/Abstract] OR unpaid[Title/Abstract]) AND (work[Title/Abstract]) AND (day[Title/Abstract] OR hour*[Title/Abstract]))))	1,198,874
**2**	"migraine disorders"[MeSH Terms] OR ‘migraine’[Title/Abstract]	46,096
**3**	#1 AND #2	1,897
**4**	("english"[Language])	31,417,195
**5**	(("2011/01/01"[Date - Publication] : "3000"[Date - Publication]))	15,419,847
**6**	#3 AND #4 AND #5	1,140
**7**	qualitative[Title/Abstract] OR interview*[Title/Abstract] OR "focus group*"[Title/Abstract] OR "study protocol"[Title/Abstract] OR editorial[Title/Abstract] OR letter[Title/Abstract] OR comment[Title/Abstract] OR "case report*"[Title/Abstract]	1,440,911
**8**	#6 NOT #7	1,018
**9**	“Europe”[MeSH] OR “United Kingdom”[tiab] OR “UK” OR “Engl*”[tiab] OR “British”[tiab] OR “London” OR “Europe*” [tiab] OR “Germany”[tiab] OR “Germ*”[tiab] OR “Berlin” OR “Hamburg”	3,910,728
**10**	#8 NOT #9	778

Table S8 - PubMed Literature Search Strategy Filtering for Publications in Spanish and Portuguese

No.	Search Terms	Hits
3	#1 AND #2 (from Table 1)	1,897
4	("english"[Language] OR “portuguese”[Language])	41

Table S9 - Embase Literature Search Strategy (English)

No.	Search Terms	Hits
1	((‘career mobility’/exp OR ‘caregiver burden’/exp OR ‘cost of illness’/exp OR ‘family leave’/exp OR ‘financial stress’/exp OR ‘retirement’/exp OR ‘return to work’/exp OR ‘social security’/exp OR ‘social welfare’/exp OR unemployment/exp)<br>OR<br>(‘Activity impairment’:ab,ti OR ‘Benefit payment’:ab,ti OR Compensation:ab,ti OR ‘Cost of illness’:ab,ti OR ‘Disability allowance*’:ab,ti OR Earning*:ab,ti OR Fiscal:ab,ti OR ‘Government transfer’:ab,ti OR ‘Hidden cost*’:ab,ti OR Income:ab,ti OR ‘Indirect cost*’:ab,ti OR ‘Indirect healthcare cost*’:ab,ti OR ‘Living allowance*’:ab,ti OR ‘Long-term disability’:ab,ti OR ‘Lost time’:ab,ti OR ‘Multifactor productivity’:ab,ti OR Pension*:ab,ti OR ‘Productive efficiency’:ab,ti OR Retire:ab,ti OR Retirement:ab,ti OR Salaries:ab,ti OR Salary:ab,ti OR ‘Social benefit*’:ab,ti OR ‘social insurance benefit’:ab,ti OR ‘Social security’:ab,ti OR ‘Tax credit’:ab,ti OR ‘Total factor productivity’:ab,ti OR ‘Transfer payment’:ab,ti OR Transportation:ab,ti OR Underemployment:ab,ti OR ‘Under-employment’:ab,ti OR Unemployment:ab,ti OR ‘Un-employment’:ab,ti OR Wage:ab,ti OR Wages:ab,ti OR Welfare:ab,ti OR WPAI:ab,ti OR ‘work life’:ab,ti OR ‘workers compensation’:ab,ti OR ‘workers comp’:ab,ti))<br>OR<br>((work*:ab,ti OR job:ab,ti OR occupation*:ab,ti OR vocation*:ab,ti OR employ*:ab,ti OR workforce:ab,ti OR ‘work-force’:ab,ti OR ‘labor force’:ab,ti OR ‘labour force’:ab,ti)<br>AND<br>(Loss:ab,ti OR productivity:ab,ti OR disability:ab,ti OR participation:ab,ti OR cessation:ab,ti OR status:ab,ti OR outcome:ab,ti OR impair*:ab,ti OR disrupt*:ab,ti OR incapacity:ab,ti OR incapability:ab,ti OR activity:ab,ti OR transportation:ab,ti OR leaving:ab,ti))<br>OR<br>((caregiver)<br>AND<br>(work*:ab,ti OR job:ab,ti OR occupation*:ab,ti OR vocation*:ab,ti OR employ*:ab,ti OR workforce:ab,ti OR ‘work-force’:ab,ti OR ‘labor force’:ab,ti OR ‘labour force’:ab,ti))<br>OR<br>(((fewer:ab,ti OR loss:ab,ti OR lost:ab,ti OR unpaid:ab,ti)<br>AND (work:ab,ti)<br>AND (hour*:ab,ti))<br>OR (‘Sick Leave’/exp OR ‘Sick Leave’:ab,ti OR ‘Sickness Absence’:ab,ti)<br>OR ‘Labor Market’:ab,ti OR ‘Labour Market’:ab,ti OR ‘RTW’:ab,ti OR ‘Out-of-pocket’:ab,ti OR ‘Productivity Loss’:ab,ti OR ‘Absenteeism’:ab,ti)	1,548,260
2	'migraine'/exp OR 'migraine'	89,617
3	#1 AND #2	4,353
4	#1 AND #2 AND ([portuguese]/lim OR [spanish]/lim)	
#5	#3 NOT #4	3,655
#6	europe' OR 'united kingdom' OR 'british' OR 'england'	
#7	#5 NOT #6	2,607
#9	(#8 NOT ('conference abstract'/it OR 'conference paper'/it OR 'conference review'/it)) AND (2011:py OR 2012:py OR 2013:py OR 2014:py OR 2015:py OR 2016:py OR 2017:py OR 2018:py OR 2019:py OR 2020:py OR 2021:py OR 2022:py OR 2023:py) AND 'human'/de AND ([adult]/lim OR [aged]/lim OR [middle aged]/lim OR [very elderly]/lim OR [young adult]/lim) AND [english]/lim	504

Table S10 - Embase Literature Search Strategy Filtering for Publications in Spanish and Portuguese

No.	Search Terms	Hits
3	#1 AND #2 (from tTble 3)	4,353
4	randomized controlled trial' OR 'case study' OR 'case report'	
#5	#3 NOT #4	3,655
#6	europe' OR 'united kingdom' OR 'british' OR 'england'	
#7	#5 NOT #6	2,607
#8	#1 AND #2 AND ([portuguese]/lim OR [spanish]/lim)	61

Figure 1 - Literature Search Flow Diagram

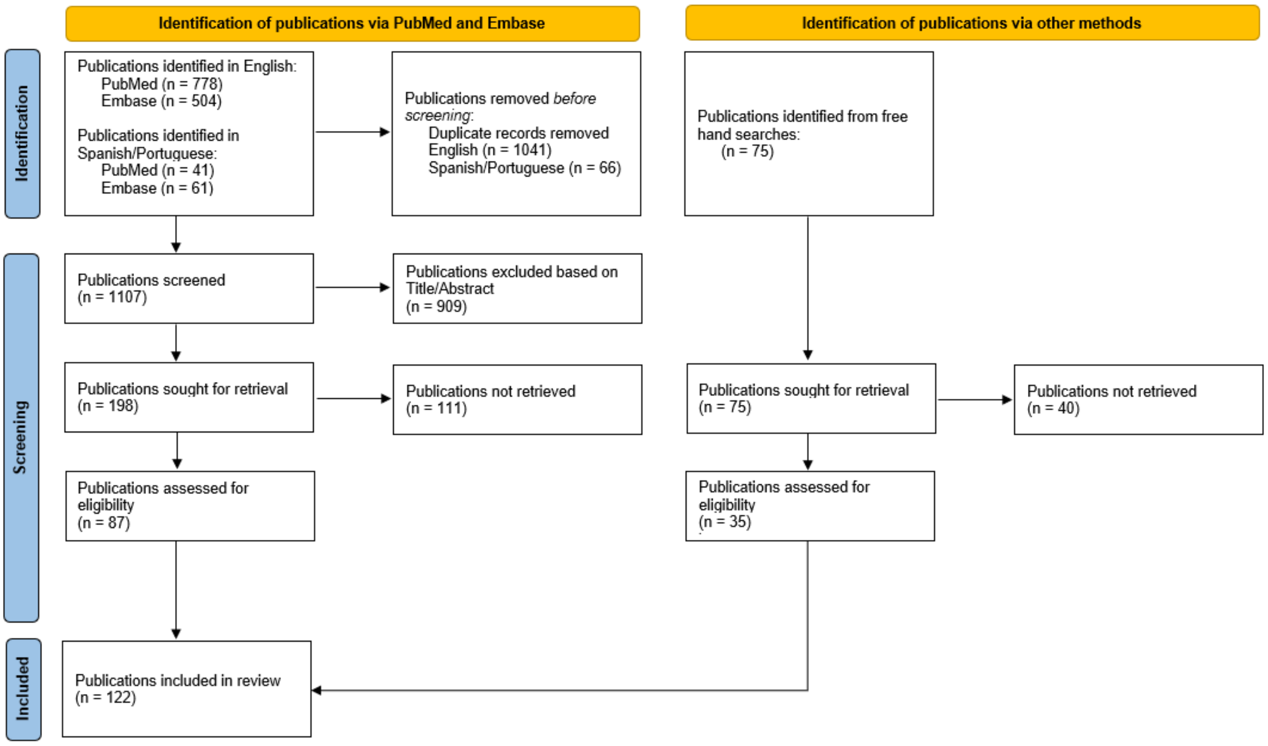

3. Impact of Migraine on Occupational Productivity and Model Application

Employment

People with migraine face challenges in maintaining labor participation due to the condition. For individuals with episodic migraine (EM, ≤14 monthly headache days [MHDs]), employment outcomes align closely with the general population, with no significant reduction in employment likelihood.[9] However, chronic migraine (CM, >14 MHDs) is associated with a reduced employment rate, with a relative risk of 0.984, indicating a slight decrease in employment likelihood compared with those with EM. The ratio of full-time to part-time employment is also impacted, with people affected by CM having a 0.963 full-time to part-time ratio relative to those with EM, suggesting a slight increase in part-time employment for those affected by chronic migraine.[10,11]

Early Retirement

Chronic migraine significantly increases the likelihood of early retirement. People with CM are modelled to have a 1.611 times higher probability of early retirement compared to those with EM. This increase in the probability of early retirement reflects the long-term burden of chronic migraine on work capacity and longevity in the workforce.[10,11]

Disability

Migraine, particularly CM, contributes substantially to disability rates. Individuals with CM are 1.802 times more likely to experience disability compared with those with EM, emphasizing the greater functional impact of CM. This metric was derived by comparing the proportions of individuals with disability in CM versus EM groups.[9]

4. Fiscal Consequences Inputs

Earnings

Earnings in the private and public sectors, as presented in **Table S7**, were estimated based on the total wage distribution and the number of employees in the public sector.[4] Annual earnings were assumed equal in employees or self-employed. Wages were adjusted using the 2023 wage index and converted to US dollars using an exchange rate of $1 = 376 Argentine pesos (ARS).

Table S11 – Average Annual Earnings by Age, Employment Type, Sector, and Sex

Age Categories	Private Sector				Public Sector			
	Part-Time ($)[a]		Full-Time ($)		Part-Time ($)		Full-Time ($)	
	Males	Females	Males	Females	Males	Females	Males	Females
18 to 24	6,281	5,021	12,561	10,042	6,413	5,655	12,825	11,310
25 to 34	8,451	6,068	16,902	12,135	7,583	6,628	15,166	13,255
35 to 44	10,621	7,114	21,243	14,229	8,754	7,600	17,508	15,200
45 to 54	11,585	6,855	23,170	13,711	10,200	8,821	20,399	17,643
55 to 64	12,549	6,596	25,098	13,193	11,645	10,043	23,291	20,086
65 and over	12,549	6,596	25,098	13,193	11,645	10,043	23,291	20,086

[a] Earnings from part-time employment were assumed to be 50% of full-time earnings.

Unemployment benefits

The unemployment benefit was set at the maximum annual amount of $4,979 for 2023.[12] This amount was converted from ARS to US dollars using the relevant exchange rate ($1 = 376 ARS).

Disability benefits

The disability benefit is a standardized annual amount of $2,362[12] and was applied uniformly across all age groups and sexes. It is calculated based on the maximum monthly unemployment benefit an individual can receive and the maximum duration of unemployment benefits, which is 12 months.[13] This benefit is granted to eligible individuals until they reach retirement age, at which point it is replaced by an old-age pension.

Old-age retirement

The old-age pension provides an annual average of $4,518[12] to individuals upon reaching the retirement age, set at 65 for males and 60 for females. This pension replaced disability benefits or other income sources, ensuring continued financial support for retirees.

Absenteeism costs

Absenteeism costs were calculated for public and private employees, as well as for self-employed individuals. The analysis also accounted for the distribution of full-time and part-time employees, and self-employed individuals. Absenteeism rates were defined for the general population and for individuals affected by EM and CM, as presented in the table below.

Table S12 – Annual Absenteeism Rates for the General and Migraine Populations

**General Population**		
**Working Days in a Year (2022)**	**Absenteeism (Days per Year) [14]**	**Absenteeism (as % of Workdays Missed)**
245	10.62	4.2%
**Migraine**		
	**Annual Workdays Missed [a]**	**Absenteeism (as % of Workdays Missed)**
**EM**	21.52	8.8%
**CM**	57.40	23.4%

Source; Ministerio de Trabajo EySS. Analysis of labor absenteeism in the national public sector: Second semester of2016.[14]

[a] Calculated based on absenteeism data for 17 European countries reported by Vo et al.[15]

Healthcare costs

Mean annual healthcare costs were calculated to be $921 for EM (≤14 MHDs) and $2,041 for CM (>14 MHDs). These values were calculated as the product of the mean annual utilization of the resources listed in **Table S13** use and the corresponding unit costs weighted by the distribution of migraine severity.

Table S13 – Mean Annual Healthcare Resource Utilization and Local Unit Costs for Migraine

	**Mean annual healthcare resource use due to migraine[a]**	**Unit costs ($, January 2024)**
General practitioner visit	5.4 [b]	11
Emergency department visit	1.6	11
Hospitalization	0.4	167
Triptan use [c]	EM = 49.4; CM = 120.9	16

Source: Souza MNP, Cohen JM, Piha T, et al. (2022)[16]; Argentina P. Local unit costs (2024).[17]

[a] Estimated as twice the difference of healthcare resource use in the past 6 months by the group with migraine and the control group.

[b] Healthcare provider visit, assumed to be priced at the cost of general practitioner visit.

[f] It was assumed that patients take triptans on 50% of their migraine days. The unit cost for triptan use represents the cost per tablet.

Table S14 – Healthcare Financing by Source Type

**Type of financing**	**Percentage of total healthcare financing (%)**
Government/compulsory schemes	29.7
Private Insurance	32.6
Out-of-pocket	37.7

Source: Argentina-Government. Gasto en salud en Argentina. Análisis para 2017-2020 (2022).[18]

Table S15 – Annual Consumer Price Index (CPI) and Wage Index

All costs used in the model were adjusted to 2023 values using the CPI and Wage Index data.

**Year**	**Consumer Price Index (CPI)**	**Wage Index**
2016	100.00	102.69
2017	124.80	129.86
2018	184.26	169.29
2019	283.44	243.40
2020	385.88	320.32
2021	582.46	501.16
2022	1134.59	981.09
2023	3533.2	2546.20

Source: INDEC. Time Series of Consumer Price Indices[19]; INDEC. Total recorded wage index, base October 31, 2016 = 100.0. [20]

5. Additional Results

The following tables illustrate the societal impact of migraine on employment income and healthcare costs, comparing individuals with migraine to those in the general population. They illustrate losses faced by the migraine population in terms of gross income and healthcare costs, highlighting the burden in the public and private workforce and healthcare funding sources. The analysis includes both a longitudinal model for the average individual over a 20-year time horizon and a cross-sectional model capturing annual costs associated with the overall population with prevalent migraine.

Table S16 - Societal Impact of Migraine on Employment Income and Healthcare Costs for an Average Individual (Longitudinal Model)

	Migraine Population	General Population	Incremental	Fiscal Impact
**Gross income from any employment**	**$138,648**	**$141,401**	**-$2,753**	**29.1% (loss)**
Private employees	$51,650	$51,744	-$95	3.4% (loss)
Public employees	$44,513	$44,594	-$81	3.0% (loss)
Self-employed	$42,485	$45,062	-$2,577	93.6% (loss)
**Total healthcare costs**	**-$6,720**	**$0**	**-$6,720**	**70.9% (loss)**
Government/compulsory schemes	-$1,996	$0	-$1,996	29.7% (loss)
Private Insurance	-$2,191	$0	-$2,191	32.6% (loss)
Out-of-pocket	-$2,533	$0	-$2,533	37.7% (loss)
**Total**	**$131,928**	**$141,401**	**-$9,473**	

Negative values indicate financial losses, while positive values reflect revenue sources for the Argentine government.

[a]All government costs and tax revenues are discounted at a rate of 3.0%.

Table S17 - Societal Impact of Migraine on Employment Income and Healthcare Costs for the Overall Population (Cross-sectional Model)

	Migraine Population	General Population	Incremental	Fiscal Impact
**Gross income from any employment**	**$24,459 M**	**$24,963 M**	**-$504 M**	**23.9% (loss)**
Private employees	$9,883 M	$9,901 M	-$18 M	3.6% (loss)
Public employees	$6,742 M	$6,754 M	-$12 M	2.4% (loss)
Self-employed	$7,834 M	$8,308 M	-$474 M	94.0% (loss)
**Total healthcare costs**	**-$1,610 M**	**$0 M**	**-$1,610 M**	**76.1% (loss)**
Government/compulsory schemes	-$478 M	$0 M	-$478 M	29.7% (loss)
Private Insurance	-$525 M	$0 M	-$525 M	32.6% (loss)
Out-of-pocket	-$607 M	$0 M	-$607 M	37.7% (loss)
**Total**	**$22,849 M**	**$24,963 M**	**-$2,115 M**	

Figure S2 - Two-Way Sensitivity Analysis of the Average Economic Burden of Migraine by Age of Onset and Time Horizon (Longitudinal Model, in Thousand US Dollars)

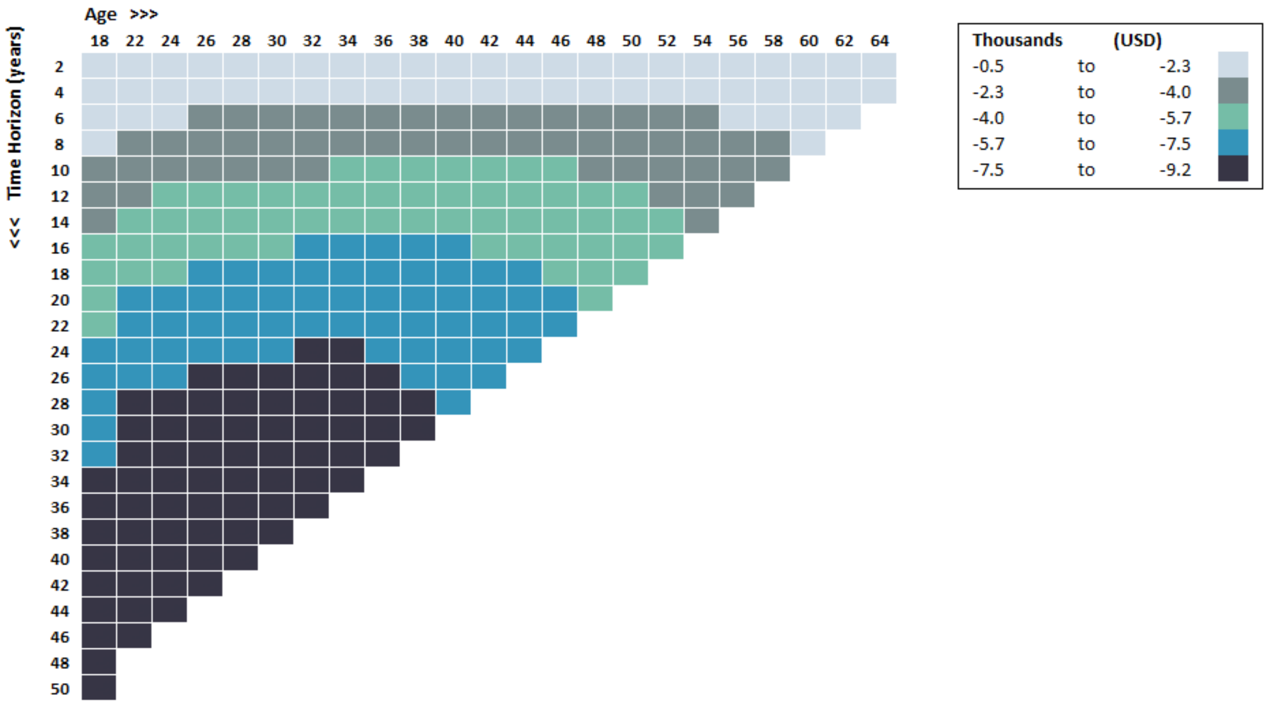

6. References

1. INDEC. Population aged 14 years and over in private households, by economic activity status, according to sex registered at birth and five-year age groups. Year 2022. Accessed 18/3/2024, https://censo.gob.ar/index.php/datos_definitivos_total_pais/
2. INDEC. Employed population aged 14 years and over in private households, by occupational category, according to sex registered at birth and five-year age groups. Year 2022. Accessed 18/3/2024, https://censo.gob.ar/index.php/datos_definitivos_total_pais/
3. INDEC. Employed Population - Number of hours worked in the week in the main occupation by age and gender. Accessed 10/10/2023, https://redatam.indec.gob.ar/argbin/RpWebEngine.exe/PortalAction?&MODE=MAIN&BASE=EAUARG&MAIN=WebServerMain.inl&_ga=2.162951932.1356699116.1696932141-178282024.1696597884
4. INDEC. Remuneration of salaried work, mixed income and labour input, by sex and age groups. Years 2016-2022. Accessed 7/2/2024, https://www.indec.gob.ar/indec/web/Nivel4-Tema-3-9-49
5. INDEC. Total population in private households. Population receiving retirement or pension according to sex registered at birth and five-year age groups. Year 2022. Accessed 4/12/2023, https://censo.gob.ar/index.php/datos_definitivos_total_pais/
6. INDEC. Total population, by sex registered at birth and femininity index, according to age. Year 2022. Accessed 18/3/2024, https://censo.gob.ar/index.php/datos_definitivos_total_pais/
7. OECD. PENSIONS AT A GLANCE 2021: ARGENTINA. 2021;
8. ANSES. Social Security Statistics (XLS format) Accessed 21/3/2024, https://www.anses.gob.ar/estadisticas-de-la-seguridad-social
9. Oliveira A, Bensenor I, Goulart A, Mercante J, Peres M. Socioeconomic and geographic inequalities in headache disability in Brazil: The 2019 National Health Survey. Article. *Headache*. 2023;63(1):114-126. doi:10.1111/head.14462
10. Blumenfeld AM, Varon SF, Wilcox TK, et al. Disability, HRQoL and resource use among chronic and episodic migraineurs: results from the International Burden of Migraine Study (IBMS). *Cephalalgia*. Feb 2011;31(3):301-15. doi:10.1177/0333102410381145
11. Payne KA, Varon SF, Kawata AK, et al. The International Burden of Migraine Study (IBMS): study design, methodology, and baseline cohort characteristics. *Cephalalgia*. Jul 2011;31(10):1116-30. doi:10.1177/0333102411410610
12. Argentina Government. *Social Security Monetary Indicators*. 2023. https://www.argentina.gob.ar/trabajo/seguridadsocial/imss
13. Iturriza AB, S. A. Unemployment Assistance and Transition to Employment in Argentina. 2021;doi:10.2139/ssrn.1158976
14. Ministerio de Trabajo EySS. *Analysis of labor absenteeism in the national public sector: Second semester of 2016.* 2016. https://www.argentina.gob.ar/sites/default/files/al_ausentismo_laboral_2_semestre_2016.pdf
15. Vo P, Fang J, Bilitou A, Laflamme AK, Gupta S. Patients' perspective on the burden of migraine in Europe: a cross-sectional analysis of survey data in France, Germany, Italy, Spain, and the United Kingdom. *J Headache Pain*. Sep 10 2018;19(1):82. doi:10.1186/s10194-018-0907-6
16. Souza MNP, Cohen JM, Piha T, et al. Burden of migraine in Brazil: A cross-sectional real-world study. Article. *Headache*. 2022;62(10):1302-1311. doi:10.1111/head.14413
17. Argentina P. Data from: Local unit costs 2024.
18. Argentina-Government. *Gasto en salud en Argentina. Análisis para 2017-2020. Secretaría de Acceso a la Salud.* 2022.
19. INDEC. Time Series of Consumer Price Indices Accessed 6-10-2023, https://www.indec.gob.ar/indec/web/Nivel4-Tema-3-5-31
20. INDEC. Total recorded wage index, base October 31, 2016 = 100.0. Accessed 6-10-2023, https://www.indec.gob.ar/indec/web/Nivel4-Tema-4-31-61
